# COPB2 is up‐regulated in breast cancer and plays a vital role in the metastasis via N‐cadherin and Vimentin

**DOI:** 10.1111/jcmm.14398

**Published:** 2019-05-22

**Authors:** Adheesh Bhandari, Chen Zheng, Namita Sindan, Namrata Sindan, Ruida Quan, Erjie Xia, Yubaraj Thapa, Dependra Tamang, Ouchen Wang, Xiaohe Ye, Duping Huang

**Affiliations:** ^1^ Department of Thyroid & Breast Surgery The First Affiliated Hospital of Wenzhou Medical University Wenzhou Zhejiang PR China; ^2^ Department of Reproductive Center The Second Affiliated Hospital of Wenzhou Medical University Wenzhou Zhejiang PR China; ^3^ Department of Pediatrics Karnali Academy of Health Sciences Chandannath Nepal; ^4^ Department of Anesthesiology Zhongda Hospital, School of Medicine Southeast University Nanjing Jiangsu PR China; ^5^ Department of Surgery Affiliated Hospital of Inner Mongolia University for Nationalities Tongliao Inner Mongolia PR China; ^6^ Department of Radiology The First Affiliated Hospital of Wenzhou Medical University Wenzhou Zhejiang PR China

**Keywords:** breast cancer, COPB2, EMT

## Abstract

Breast cancer (BC) is a common malignant tumour for the adult female and its relative incidence has increased continuously in recent years. The primary molecular mechanisms of breast tumourigenesis remain unclear. With the sequencing technology, we found that coatomer protein complex subunit beta 2 (COPB2) gene is overexpressed in breast cancer tissues. However, the biological function of COPB2 in BC has yet to be determined. This current research demonstrates, significant up‐regulation of COPB2 in tissues of breast cancer while comparing the adjacent normal tissue both invalidated cohort and TCGA cohort. Up‐regulated expression of COPB2 was correlated with lymph node metastasis (LNM) and oestrogen receptor (ER) in the TCGA cohort and a high level of COPB2 was associated with age and lymph node metastasis in the validated cohort. Besides, logistic analysis illustrated in BC patient COPB2 expression, tumour size, age, ER and disease stage were independent high‐risk factors of LNM. Loss of function experiments revealed that down‐regulation of COPB2 could inhibit capacities of proliferation and cell invasion in MDA‐MB‐231 and BT‐549 cell lines. Moreover, underexpression of COPB2 could decrease the EMT‐related protein N‐cadherin and vimentin which may lead to cell invasion. This current research provides new shreds of evidence that COPB2 overexpression shows significant character in the progression of breast cancer. To best of our knowledge, our findings indicated that COPB2 was vital oncogene which was associated with breast cancer.

## 1 INTRODUCTION

Breast cancer (BC) is one of the widespread malignant cancer in women with 268 670 newly projected identified cases and 41,400 expected deaths in the United States in 2018.^1^ Significant improvement has been made in recent decades and breast cancer can be cured with surgery, endocrine therapy, cytotoxic or targeted therapies. Throughout the past years, emerging research has been done in discovering the underlying mechanisms of BC.^2^, ^3^, ^4^ However, the therapeutic effect is not satisfactory.^5^, ^6^, ^7^ Due to the development of medical technology and the perfection of social welfare, part of the breast cancer patient can be treated by surgery, endocrine therapy, chemotherapy or targeted therapies. However, the effect of those treatments is not always gratifying.^8^, ^9^ Oestrogen receptor (ER)‐positive (or luminal) tumours represent around 60% to 75% of all breast cancers and endocrine therapy is highly effective and appropriate for nearly all females with tumours. Unfortunately, some subtype of breast cancers often becomes resistant to endocrine treatment, relapse and becomes fatal, underscoring the need for the identification of novel molecular biomarkers that can predict the progression and prognosis of this disease.

The coatomer protein complex subunit beta 2 (COPB2, also known as Beta‐Cop, P102 or Coatomer Protein Complex Subunit Beta Prime) gene encodes a 102‐kDa protein and maps to chromosome 3q23. COPB2 is a subunit of the Golgi coatomer complex, a cytosolic protein complex that composes the coat of non‐clathrin‐coated vesicles and is crucial for vesicular trafficking and Golgi budding. Recently, COPB2 is involved in tumourigenesis in numerous kinds of cancer including lung adenocarcinoma and prostate cancer.^10^, ^11^ However, the clinical significance of COPB2 in patients with breast cancer remains unknown.

In this study, we performed quantitative real‐time PCR (qRT‐PCR) in 56 breast cancer tissues and its adjacent normal tissue to validate the results of sequencing technology. Subsequently, we used the small interfering RNA (siRNA) to knockdown the expression of COPB2 in two types of breast cancer cell lines (MDA‐MB‐231 and BT‐549). Furthermore, the expression of COPB2 in breast cancer tissues and adjacent non‐cancerous tissues was both analysed in a TCGA cohort and local cohort. In the TCGA cohort, logistic analysis illustrated in BC patient COPB2 expression, tumour size, age, oestrogen receptor and disease stage were independent high‐risk factors of LNM.

Our study aimed to determine the relationship between COPB2 expression and the role of COPB2 in the proliferation and metastasis in breast carcinoma.

## 2 MATERIALS AND METHODS

### 2.1 Patients and samples

Fifty‐six breast cancer tissues and 56 paired adjacent non‐cancerous tissues were gained from the Department of Thyroid and Breast Surgery, The First Affiliated Hospital of Wenzhou Medical University. Samples were frozen in liquid nitrogen immediately after surgical removal and stored at −80°C until further RNA detection. All patient's information was collected under the protocols approved by the institutional review board of the Ethics Committee of the First Affiliated Hospital of Wenzhou Medical University (approval no. 2012‐57).

### 2.2 The Cancer Genome Atlas (TCGA) data

Public data of COPB2 gene expression for breast cancer patients and their clinical information were downloaded from the Cancer Genome Atlas Project (TCGA) (https://tcga-data.nci.nih.gov/tcga/). A total of 1091 cases of breast cancer patients with complete clinical features were selected and 228 pairs of patients with normal tissue matching were selected.

### 2.3 RNA extraction and real‐time quantitative polymerase chain reaction (RT‐qPCR)

According to the manufacturer's instructions (Invitrogen, USA), RNA from cells was isolated by TRIZOL reagent (Invitrogen, USA) according to the manufacturer's protocol. The purity of RNA was examined at 260/280 nm by spectrophotometry (Thermo, San Jose, CA, USA). All RNA samples were reverse transcribed (Toyobo, Osaka, Japan). Real‐time reactions were run and analysed by Real‐Time PCR system (Applied Biosystems 7500). The expression level of GAPDH mRNA was used for the normalization. The sequences of the primers used were:

COPB2, forward 5'‐ CTTCCTGTTCGAGCTGCAAAG‐3' and reverse 5'‐ CACTCTAATCTGCATGTCATCCG −3'. GAPDH forward: 5'‐GTCTCCTCTGACTTCAACAGCG‐3' and reverse: 5'‐ACCACCCTGTTGCTGTAGCCAA‐3'.

The comparative CT method (∆∆CT) method was used to evaluate the relative quantification of COPB2. Each sample was performed in triplicate.

### 2.4 Cell cultures and growth conditions

We had used MDA‐MB‐231, BT‐549, SK‐BR‐3, BT‐474, MCF‐7 and MCF‐10A cell lines to perform the experiment in this study. All cell lines were purchased from Shanghai Cell Biology, Institute of the Chinese Academy of Sciences (Shanghai, People's Republic of China). DMEM (Gibco, Grand Island, NY，USA) was supplemented with 10% FBS (Gibco, Grand Island, NY, USA) to culture MDA‐MB‐231, MCF‐7, SK‐BR‐3 cell lines. Roswell Park Memorial Institute‐1640 medium (Gibco, Grand Island, NY, USA) was mixed with 10% FBS (Gibco) to culture BT‐549 and BT‐474 cell lines. DMEM‐F12 (Gibco) was supplemented with 100 μg/mL of streptomycin, 100 U/mL of penicillin, 20 ng/mL of epidermal growth factor (EGF), 2 mmol/L of l‐glutamine and 10% FBS (Gibco) to culture MCF‐10A cell line. All cell lines were incubated in a standard cell culture incubator (Thermo, Waltham, MA, USA) at 37°C with 5% CO_2_.

### 2.5 RNA interference

siRNA for COPB2 was obtained from Shanghai Gene Pharma (Shanghai, China) for cell interference. The sequences of the COPB2 are as follows: COPB2 (Sense‐1: CCCAUUAUGUUAUGCAGAUTT; Antisense‐1: AUCUGCAUAACAUAAUGGGTT; Sense‐2: GCAGAUGACCGUCUUGUUATT; Antisense‐2: UAACAAGACGGUCAUCUGCTT); Negative controls (si‐NC) in our experiments were scramble sequences which were randomly added with no any target sequence tracks. According to the manufacturer's protocol, the Lipofectamine RNA iMAX transfection reagent (Thermo Fisher Scientific) was mixed with siRNA to transfect MDA‐MB‐231 and BT‐549 cell lines. Briefly speaking, about 120,000 BC cells were seeded into 6‐well plates. The siRNA concentrations for MDA‐MB‐231 and BT‐549 were produced at 100 nmol/L each plate. After 48 hours, transfected cells were harvested for subsequent RNA expression analysis. All knockdown experiments were conducted in triplicate.

### 2.6 Cell invasion and migration assay

To detect cell capacity of migration and invasion, we used cell culture chambers according to the manufacturer's instructions (Corning Costar Corp., Cambridge, MA, USA). About 40 000/well of si‐NC or si‐COPB2 cells were transferred into the upper chamber and the lower chamber was filled with 600 mL of medium containing 20% FBS.

Then, the chamber was placed in the incubator in the above environment (37°C with 5% CO_2_). After 24 hours, the chamber was carefully removed and fixed with 4% paraformaldehyde and 0.4% crystal violet was used for staining the membrane in 15 minutes. Five random fields of view were selected for analysis and images were captured by a microscope at 20 × magnification.

### 2.7 Cell proliferation assay

For the CCK‐8 proliferation assay, about 1500/well of MDA‐MB‐231 and BT‐549 were seeded into 96‐well plates. Subsequently, all cells were transfected with target siRNA. After 24, 48, 72 and 96 hours, we assessed each well cell proliferation by measuring the absorbance of 450 nm.

For colony forming assay, about 1500/well of MDA‐MB‐231 and BT‐549 were seeded into 96‐well plates and maintaining it in RPMI1640 or DMEM supplied with 10% FBS for 14 days and 0.4% crystal violet was used for staining the membrane in 15 minutes. All images were captured by the microscope camera. Above experiments were performed in triplicate.

### 2.8 Apoptosis detection analysis

Annexin‐V‐FITC apoptosis kit (Nanjing KeyGen Biotech. Co., Ltd., Nanjing, China) was used to determine the proportion of apoptotic cells. We collected the transfected cells in 6‐plate and centrifuged cells at 1000 rpm for 5 minutes three times. After each centrifuging, we resuspended cells with 1 mL PBS. Subsequently, we mixed resuspended cells in 500 μL binding buffer and 5 μL Annexin V‐FITC. All experiment data were analysed by flow cytometry.

### 2.9 Protein extraction and Western blot analysis

The collected cells were cleaved in cell lysis buffer (Beyotime, China) to obtain whole‐cell lysates. Protein concentrations were measured using bicinchoninic acid assay (BCA). We used 10% SDS‐PAGE on the gel to separate total cell lysate proteins. Then all samples were electro‐transferred onto PVDF membranes. For blocking the blots, the TBST mixed 5% non‐fat milk was used for 2 hours at room temperature. Next, the blots were probed with polyclonal antibody at 4°C overnight. Finally, the blots were incubated with the secondary antibody (goat anti‐rabbit IgG conjugated with HRP, Abcam, CA) for 1 hour at room temperature. Primary antibodies were as follows: N‐cadherin, vimentin, human β‐actin (all Cell Signaling Technology, Danvers, MA).

### 2.10 Statistical analysis

Expression difference was analysed by Paired *t*‐test. Clinicopathological characteristics were assessed by Chi‐square test. Cox regression analyses were used to evaluate the association between COPB2 and LNM. Differences were considered to be statistically significant at *P* < 0.05. Statistical analyses were achieved using SPSS 23.0 statistical software package (SPSS, Inc, Chicago, IL) and GraphPad Prism version 7.01 (GraphPad Software, Inc, La Jolla, CA).

## 3 RESULTS

### 3.1 COPB2 was significantly up‐regulated in breast cancer

**Figure 1:**
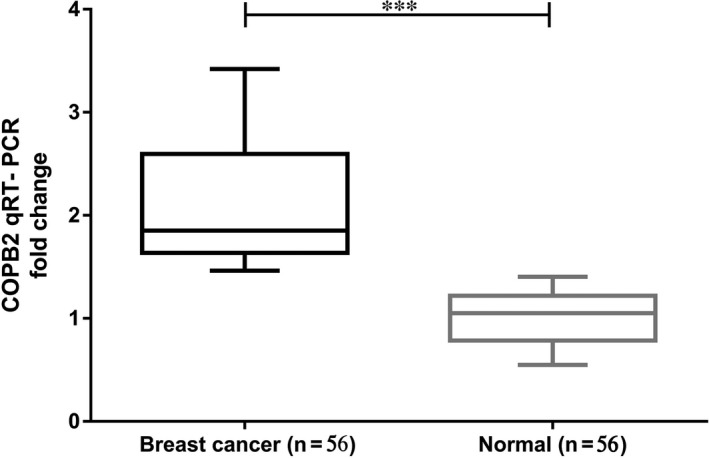
qRT‐PCR results of COPB2 mRNA expression levels in human samples. Relative mRNA levels of COPB2 between 56 breast cancer tumours (Tumour) and 56 paired adjacent normal tissues (Control) were presented. Median with interquartile range is marked. A significant difference was revealed between the two groups using the Wilcoxon Signed Ranks Test (*** *P *＜ 0.001)

**Figure 2:**
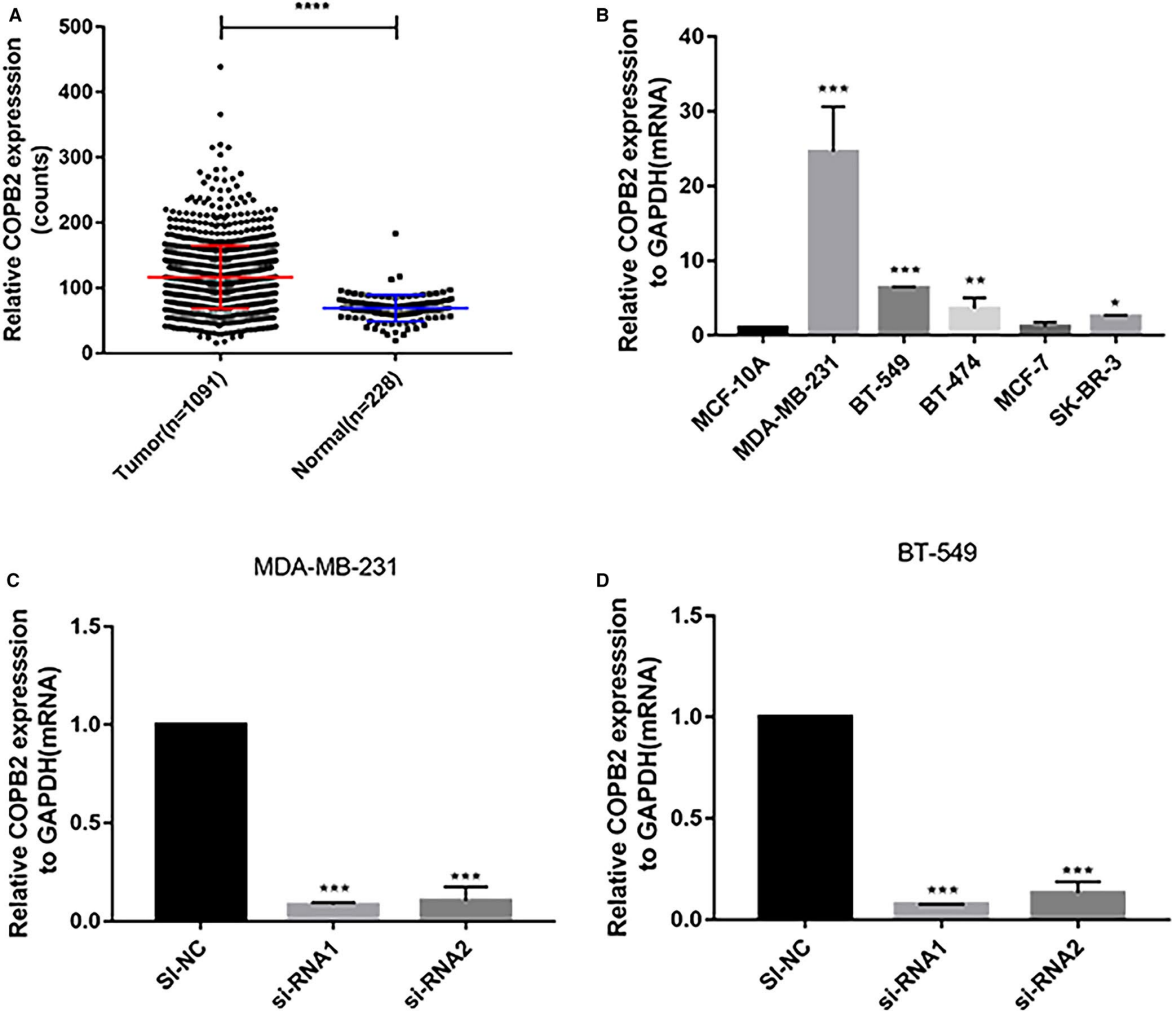
The expression level of COPB2 in breast cancer cell lines. A, COPB2 expression in breast cancer in the TCGA cohort. A total of 1091 cases of breast cancer patients and 228 pairs of patients with normal tissue matching with complete clinical features were selected. B, The relative expression of COPB2 (compared with the GAPDH gene) was examined via RT‐qPCR. C and D, The relative expression of COPB2 (compared with the GAPDH gene) in MDA‐MB‐231 and BT‐549. The expression of COPB2 in si‐RNA1 and si‐RNA2 group was significantly lower expression than the corresponding si‐NC group. (*P < 0.05；**P < 0.01； ***P < 0.001)

Through the whole transcriptome sequencing of 15 pairs of the breast tumour and adjacent normal tissues, we found that the expression level of COPB2 in breast cancer tissues was significantly higher than that in normal tissues (Table 1). To validate the result of sequencing, we selected another 56 paired breast cancer tissues and adjacent non‐cancerous tissues to examine the expression of COPB2 mRNA by qRT‐PCR. As shown in Figure 1, the expression of COPB2 mRNA expression was significantly up‐regulated in breast cancer tissues compared with the adjacent normal tissues (*P* < 0.001). In order to further investigate the dysregulated expression of COPB2, RNA sequencing data of breast cancer were extracted from the TCGA database which contained with 1091 cases of BC patients and 228 pairs of patients with normal tissue. Consistently, the expression of COPB2 mRNA was significantly up‐regulated in BC tissues (Figure 2A, *P* < 0.001). These results suggested that COPB2 gene may act as a potential tumour oncogene in breast cancer patients.

**Table 1:** The expression of COPB2 gene in 15 cases of breast cancer was greater (Up‐regulated) than that in normal tissue by whole transcriptome sequencing

Symbol	RN‐expression	RT expression	Log 2 ratio (RT/RN)	RT/RN
COPB2	3686	11173	0.481614	Up
COPB2	4170	8320	0.299987	Up
COPB2	4603	5794	0.099938	Up
COPB2	3158	10759	0.53236	Up
COPB2	2994	8257	0.44057	Up
COPB2	3063	4663	0.182518	Up
COPB2	2111	5380	0.406294	Up
COPB2	1427	3804	0.425817	Up
COPB2	1649	5776	0.544407	Up
COPB2	5044	5340	0.024766	Up
COPB2	3595	9705	0.431297	Up
COPB2	3146	4919	0.194118	Up
COPB2	3017	6008	0.299155	Up
COPB2	2924	15981	0.737627	Up
COPB2	2716	4545	0.223604	Up

Abbreviations: RN, RNA normal tissues; RT, RNA tumour tissues.

### 3.2 The relationship between COPB2 expression and clinicopathologic characteristics in breast cancer

To investigate whether COPB2 was associated with the development and progression of breast cancer, a total of 1091 breast cancer patients were divided into low and high COPB2 expression groups according to the median value. As shown in Table 2, the human oestrogen receptor (ER) expression (*P* = 0.038) and lymph node metastasis (*P* = 0.035) were significantly related to the expression of COPB2. However, there were no differences between the two groups in terms of tumour size, age, distant metastasis, clinical stage and progesterone receptor (PR). In our validated cohort, we found that higher COPB2 expression group had more lymph node metastasis (*P* = 0.033) and younger age (*P* = 0.007) than lower COPB2 expression group (Table 3).

**Table 2:** The relationship between COPB2 expression and clinicopathologic characteristics in the TCGA cohort

Characteristic	High COPB2 (n = 545)	Low COPB2 (n = 546)	*X^2^*	*P*
Age			0.33	0.566
<60	285	295		
≥60	260	251		
Tumour size			2.126	0.145
≤2 cm	129	150		
>2 cm	415	394		
Lymph node metastasis			4.468	0.035*
Present	295	262		
None	239	275		
Distant metastasis			2.852	0.091
Yes	10	19		
No	535	527		
Clinical stage (AJCC7)			0.008	0.929
I‐II	396	404		
III‐IV	133	134		
Progesterone receptor			2.17	0.141
Positive	360	335		
Negative	161	182		
Oestrogen receptor			4.326	0.038*
Positive	418	386		
Negative	105	132		

* *P* < 0.05.

**Table 3:** The relationship between COPB2 and clinicopathologic characteristics in the validated cohort

Clinicopathologic characteristics	High expression (%)	Low expression (%)	*X* ^2^	*P*
Age			7.179	0.007*
≤60	20 (71.4%)	10 (35.7%)		
＞60	8 (28.6%)	18 (64.3%)		
Tumour size			1.5	0.472
<2 cm	14 (50.0%)	18 (64.3%)		
2‐5 cm	10 (35.7%)	6 (21.4%)		
>5 cm	4 (14.3%)	4 (14.3%)		
Lymph node metastasis			4.571	0.033*
No	10 (35.7%)	18 (67.3%)		
Yes	18 (67.3%)	10 (35.7%)		
Clinical stage			0.084	0.771
I‐II	19 (67.9%)	20 (71.4%)		
III‐IV	9 (32.1%)	8 (28.6%)		

* *P* < 0.005.

### 3.3 High expression levels of COPB2 were correlated with LNM in breast cancer

In order to assess the effects of COPB2 expression on the prognosis of breast cancer, we analysed follow‐up data of the TCGA cohort. As showed in Table 4, Univariate cox regression analysis indicated that the high COPB2 expression was independent high‐risk factor as well as tumour size(≥2 cm), old age (≥60), ER status and clinical stage (HR = 1.296, 95% Confidence Interval [CI] 1.019‐1.647, *P* = 0.035; HR = 2.383, 95%CI 1.793‐3.168, *P* < 0.001; HR = 0.631, 95%CI 0.495‐0.804, *P* < 0.001; HR = 1.684, 95%CI 1.255‐2.261, *P* = 0.001; HR = 48.887, 95%CI 23.756‐96.503, *P* < 0.001 respectively). Meanwhile, multivariate logistic analysis validated that COPB2 expression (OR = 1.348, 95% CI = 1.001‐1.815, *P* = 0.049), tumour size (OR = 1.581, 95% CI = 1.133‐2.205, *P* = 0.007), age (OR = 0.494, 95% CI = 0.365‐0.670, *P* < 0.001), ER status (OR = 2.027, 95% CI = 1.403‐2.929, *P* < 0.001) and clinical stage (OR = 53.116, 95% CI = 25.591‐110.246, *P* < 0.001) were significant high‐risk factors of LNM by using all parameters (Table 5). These results showed that high‐COPB2 expression might influence the ability of migration of breast cancer cells.

**Table 4:** Univariate Cox regression analysis for risk factors associated with LNM

Factor	HR	95% CI	*P*–value
COPB2 expression (high, vs low)	1.296	1.019‐1.647	0.035*
Tumour size (<2 cm vs ≥ 2 cm)	2.383	1.793‐3.168	<0.001*
Age, years (<60 vs ≥ 60)	0.631	0.495‐0.804	<0.001*
PR (Positive vs. Negative)	1.285	0.989‐1.669	0.06
ER (Positive vs. Negative)	1.684	1.255‐2.261	0.001*
HER‐2 (Positive vs. Negative)	1.125	0.818‐1.546	0.469
Clinical stage (AJCC7)	48.887	23.756‐96.503	<0.001*

HR = Hazard ratio; CI = confidence intervals.

* *P* < 0.005.

**Table 5:** Multivariate Cox regression analysis for risk factors associated with LNM

Factor	HR	95% CI	*P*–value
COPB2 expression (high, vs low)	1.348	1.001‐1.815	0.049*
Tumour size (<2 cm vs ≥ 2 cm)	1.581	1.133‐2.205	0.007*
Age, years (<60 vs ≥ 60)	0.494	0.365‐0.670	<0.001*
ER (positive vs negative)	2.027	1.403‐2.929	<0.001*
Clinical stage (AJCC7)	53.116	25.591‐110.246	<0.001*

HR = Hazard ratio; CI = confidence intervals.

* *P* < 0.005.

### 3.4 The expression level of COPB2 in breast cancer cell lines

Whether COPB2 overexpressed in BC cell lines, we also estimated the relative COPB2 expression level both in several BC cell lines and normal breast cell lines (MCF‐10A) via using RT‐qPCR. The results showed that COPB2 gene was overexpressed in MDA‐MB‐231, BT‐549, SK‐BR‐3 and BT‐474 comparing with MCF‐10A (Figure 2B). Therefore, we selected the relative higher expression cell line MDA‐MB‐231 and BT‐549 for further experiment. Next, we designed two effective siRNA to knock down the expression of COPB2 in those cell lines and the resulting expression was evaluated using RT‐qPCR analysis. (Figure 2C and 2).

### 3.5 COPB2 gene knockdown inhibits breast cancer cell proliferation and promotes apoptosis

**Figure 3:**
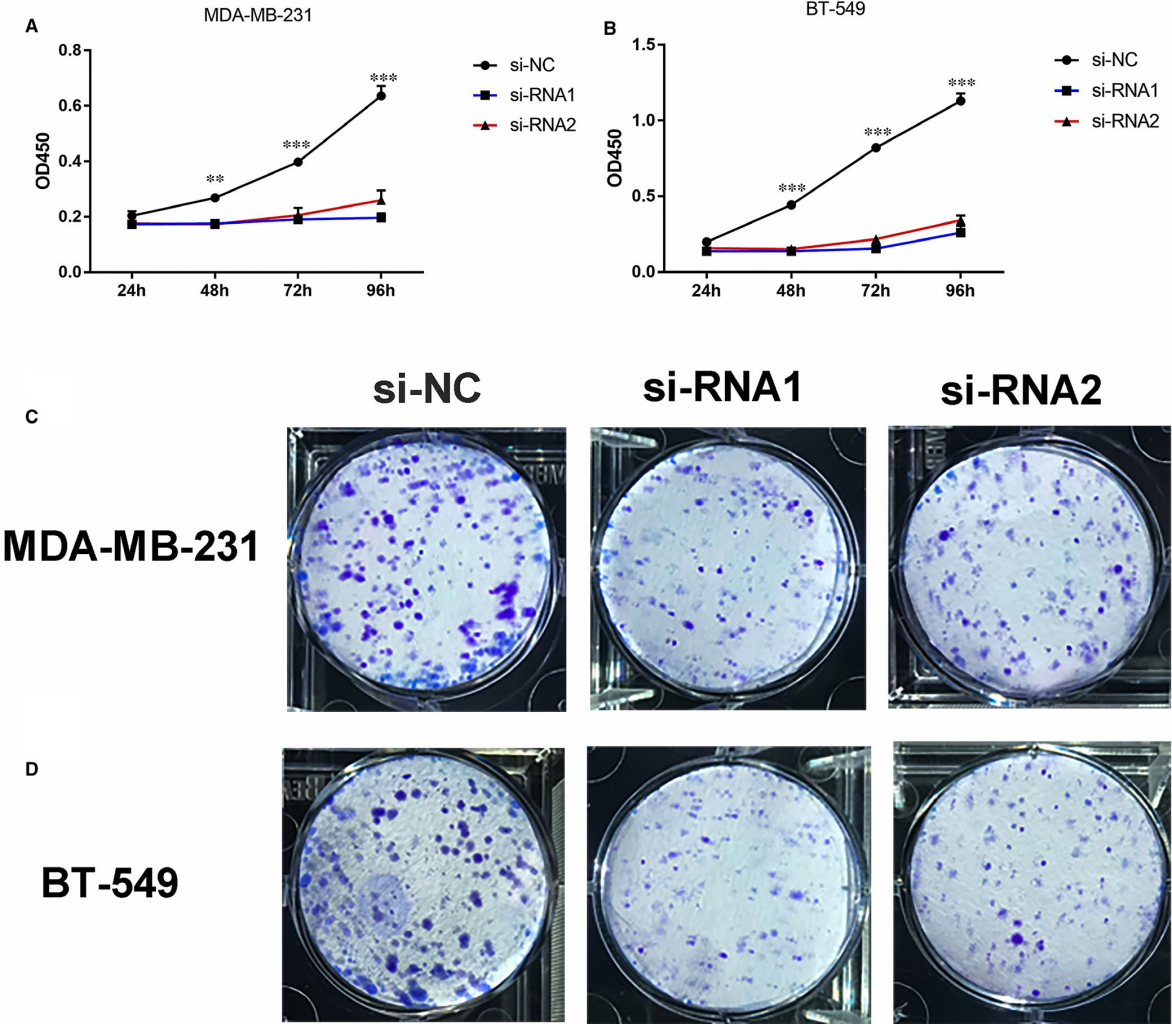
COPB2 down‐regulation can inhibit BC cell proliferation. A and B, CCK‐8 assay：MDA‐MB‐231 and BT‐549 cell lines transfected with si‐RNA1 or si‐RNA2 were cultured for 1‐4 d and measuring the absorbance of the wells at 450 nm. C and D, Colony formation: si‐RNA1 and si‐RNA2 significantly restrain cell proliferation in BC cell lines. (*P < 0.05；**P < 0.01； ***P < 0.001)

Considering COPB2 was overexpressed in above BC cell lines, we hypothesized this gene might be playing a vital role in BC tumourigenesis and progression. Cell proliferation and colony formation assays were then performed. The results revealed that down‐regulation of COPB2 could effectively inhibit BC cell lines proliferation and colony formation compared with the si‐NC group (Figure 3A‐D). Cell proliferation was significantly suppressed in BC cell transfected by si‐RNA1 and si‐RNA2.

**Figure 4:**
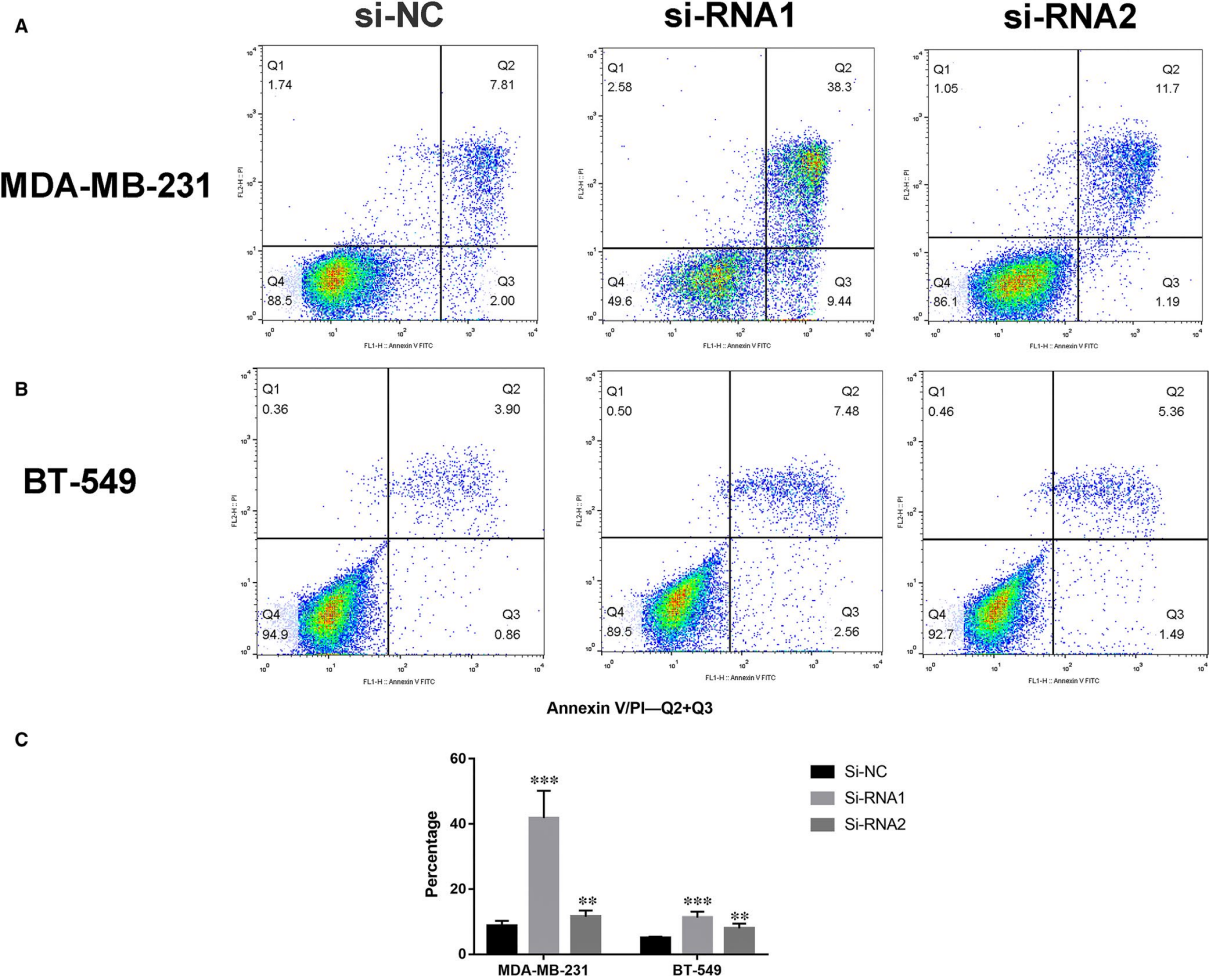
COPB2 knockdown can induce cell apoptosis of BC cell. A and B, MDA‐MB‐231 and BT‐549 cell lines transfected with si‐NC or si‐RNA1 or si‐RNA2. Knocking down COPB2 could increase apoptotic cell death in BC cells. C Histogram of the FACS data. The columns represent the mean of death cell numbers from at least three independent experiments. (**P < 0.05, ***P < 0.01 in comparison with si‐NC using student's *t*‐test.)

To further validate whether COPB2 influences breast cell tumourigenesis, we performed apoptosis assays. The si‐RNA1 or si‐RNA2 can induce cell apoptosis in the MDA‐MB‐231 and BT‐549, especially late‐stage apoptotic cells, compared with that in control cells (Figure 4A‐C).

### 3.6 Down‐regulation of COPB2 inhibits breast cancer cell migration and invasion

**Figure 5:**
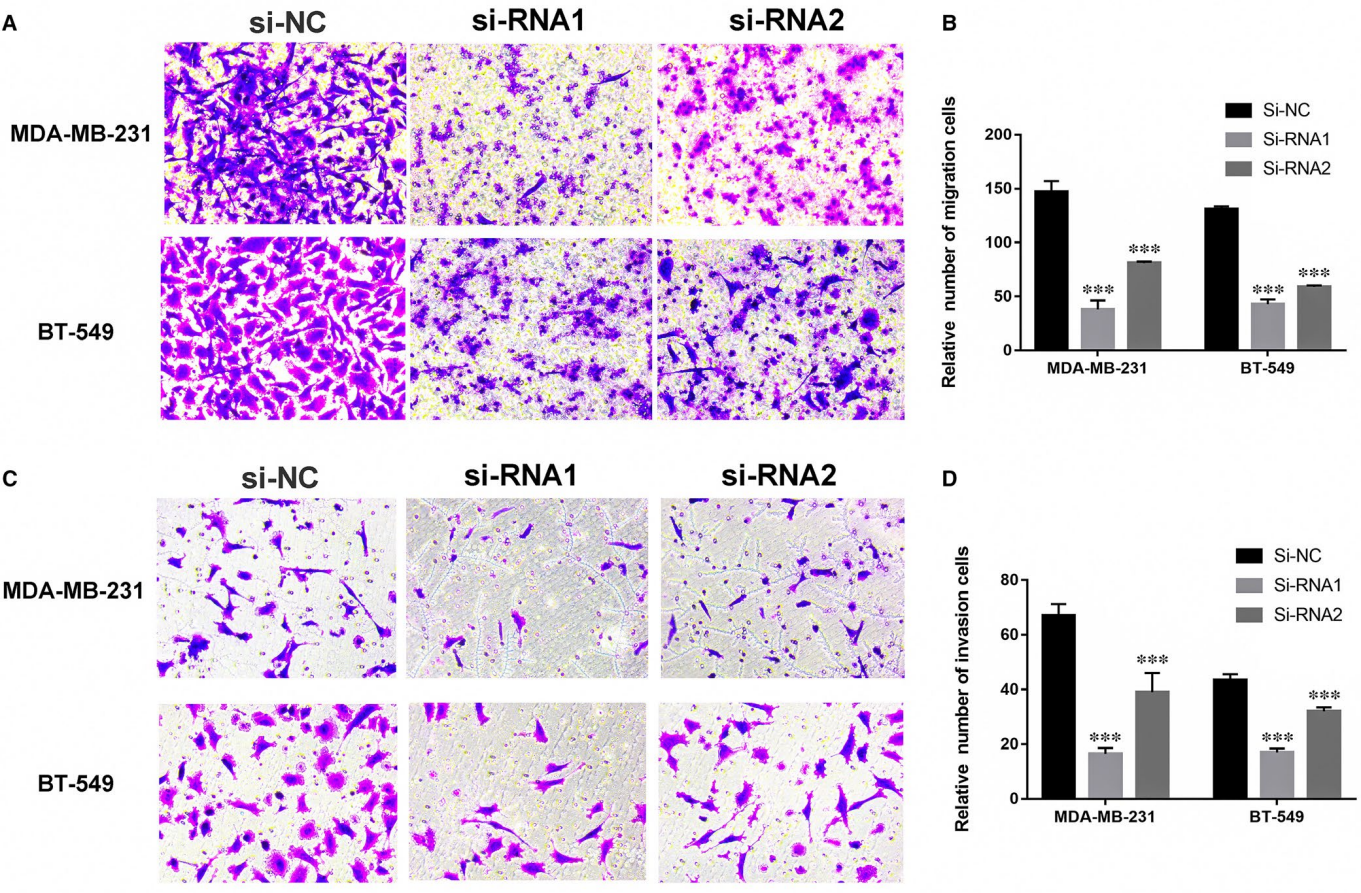
Down‐regulation of COPB2 inhibits breast cancer cell migration and invasion. A and C, In MDA‐MB‐231 and BT‐549, migration and invasion assays in si‐COPB2 cell lines and their corresponding control cells. B and D, The columns represent the mean of death cell numbers from at least three independent experiments. (**P < 0.05, ***P < 0.01 in comparison with si‐NC using student's *t*‐test.)

In order to validate the function of COPB2 in the breast cancer cell, we performed the cell migration and invasion assay. As expected, underexpression of COPB2 can significantly inhibit breast cancer cell's capacities of migration and invasion compared with the si‐NC control group (Figure 5A‐D).

### 3.7 The COPB2 gene regulates epithelial‐mesenchymal transition (EMT)

**Figure 6:**
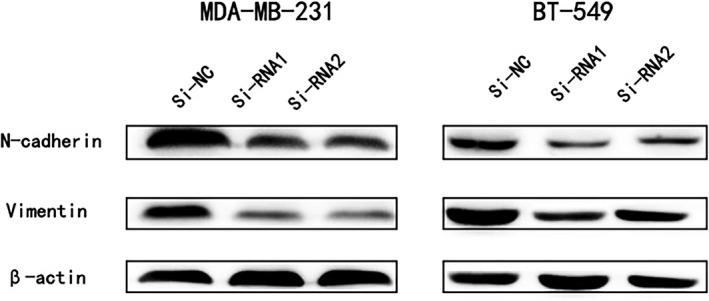
The COPB2 gene regulates epithelial‐mesenchymal transition (EMT). The influence of COPB2 expression on the levels of N‐cadherin, vimentin in MDA‐MB‐231 and BT‐549 (transfected with si‐RNA1, 2 or si‐NC) by western blot

Accumulating researches have increasingly recognized epithelial‐mesenchymal transition (EMT) as a vital process during cancer cell metastasis.^12^, ^13^, ^14^ As shown in Figure 6, the expression of vimentin and N‐cadherin was deceased in si‐COPB2 cell lines. Those result suggested that COPB2 may promote BC cell metastasis by influencing EMT.

## 4 DISCUSSION

Breast cancer is the most frequent malignant cancers which are diagnosed and it is also one of the main reasons for death worldwide.^1^, ^14^ In China, the occurrence rate of breast cancer has soared obviously in recent decades.^15^ Oestrogen receptor (ER), progesterone receptor (PR) and human epidermal growth factor receptor 2 (HER2) are common breast cancer biomarkers and the classification of breast cancer is mainly based on them. Being a heterogeneous disease, the information provided by the hormone receptor status is always not enough for proper treatment.^16^ Although much progress in breast cancer research has been made, the specific molecular mechanisms of breast cancer remain unknown. High‐throughput sequencing analysis of genetic alterations, an advanced sequencing method, have been utilized to explore its molecular mechanisms.

COPB2 is the main representative of β'‐COP in the COPI complex, which is built up of seven subunits: α‐COP (COPA), β‐COP, γ‐COP, δ‐COP, β'‐COP (COPB), ε‐COP and ξ‐COP (COPZ) and COPB2 were among the definite features in the protein collaboration network.^17^ Gordon et al^18^ showed that mRNA expression was increased in mesothelioma tissues, which was explained by one of the subunits in COPI along with large scale transcription profile. Likewise, Sudo et al^19^ found the involvement of COPA and COPB2 during cellular growth as well as during apoptosis of mesothelioma cells in vitro.

COPA and COPB have the amino acid repeat of tryptophan and aspartic acid that has been connected to up‐regulation of the cell cycle, signal transduction as well as apoptosis.^20^ Mi et al^21^ showed that in prostate cancer tissues, COPB2 gene was highly up‐regulated. Also, PC3 cells were significantly proliferated which lead to the modulation of colony formation and cell death.

With the development of technique, sequencing analysis has been applied to understand its molecular mechanisms comprehensively. Current study reported that COPB2 was overexpressed which in turn was associated with human breast cancer promotion. Our study specifies new ideas and shreds evidence that COPB2 overexpression shows its significant roles in breast cancer progression. Clinical results in breast cancer patients and possible coefficient of correlation with COPB2, however, remain unidentified.

Here, we found that COPB2 was significantly up‐regulated in a large cohort of human breast cancer tissues and that COPB2 levels significantly linked with the clinical characteristics of BC, including oestrogen receptor (ER) and BC lymph node metastasis. Similarly, the multivariate logistic analysis revealed that COPB2 expression, tumour size, age, ER status and clinical stage were significant high‐risk factors of LNM. This signifies that COPB2 can predict the probability of LNM in breast cancer patients justifying additional investigations.

To demonstrate the function of COPB2 in breast cancer, we examined 56 matched BC tumour tissue and adjacent normal tissues. Though RT‐qPCR, the mRNA expression level of COPB2 was evaluated in a local cohort and different breast cancer cell lines. In vitro experiments illustrated that knockdown of COPB2 can effectively inhibit cell proliferation and apoptosis. Moreover, down‐regulation of COPB2 also can restrain the BC cell abilities of migration and invasion. Finally, we detected the protein expression of EMT‐related molecules by immunoblotting and found low N‐cadherin and vimentin expressions in the si‐COPB2 cell lines.

In spite of our interesting discovery, there are still some limitations to the study.

First, more tumour samples are needed to analyse the correlation of COPB2 with clinical parameters and confirm its role in BC metastasis. Besides, in vivo experiments and precise cellular mechanisms for COPB2 are required to be further studied.

## 5 CONCLUSIONS

In short, our research shows the increased expression of COPB2 in breast cancer in humans when compared to normal tissues and COPB2 may predict breast cancer metastasis and also it might be an independent molecular marker.

## CONFLICT OF INTEREST

The authors have no conflicts of interest to disclose.

## **AUTHORS**'** CONTRIBUTION**

Adheesh Bhandari wrote the manuscript. Adheesh Bhandari, Erjie Xia and Zheng Chen did the main experiments. Namita Sindan, Namrata Sindan, Ruida Quan and Xiaohe Ye collected and analysed the raw data. Yubaraj Thapa and Dependra Thapa helped to revise the article. Ouchen Wang and Duping Huang designed the whole work.

## ETHICAL APPROVAL AND CONSENT TO PARTICIPATE

Ethical approval for this study was obtained from the Ethics Committee of the First Affiliated Hospital of Wenzhou Medical University.

## CONSENT FOR PUBLICATION

Written informed consent was issued by the patients for the publication of this research and accompanying images. A copy of the written consent is ready for review by the Editor in Chief of this journal. Consent for publication was obtained from all participants.

## DATA AVAILABILITY STATEMENT

The data sets supporting the conclusions of this study are included in this article and its, additional images. Raw data are available on the main electronic data storage system of First Affiliated Hospital of Wenzhou Medical University and access can be provided upon request to the authors.
